# Knowledge, attitudes, and practices regarding the postoperative management and TSH suppression therapy among patients with thyroid cancer

**DOI:** 10.3389/fonc.2025.1441726

**Published:** 2025-03-11

**Authors:** Yongkang Wu, Zhi Zhang, Wenyi Qin, Weijie Chen, Tuo Xu

**Affiliations:** Department of Vascular and Thyroid Surgery, the Affiliated Hospital of Guangdong Medical University, Zhanjiang, China

**Keywords:** knowledge, attitude, practice, thyroid cancer, thyroid-stimulating hormone, cross-sectional study

## Abstract

**Background:**

Patient adherence to thyroid-stimulating hormone (TSH) suppression therapy is crucial for optimizing prognosis.

**Methods:**

This study aimed to examine the knowledge, attitude, and practice (KAP) toward TSH suppression therapy among patients. This cross-sectional study was conducted at our Hospital between March 2023 and October 2023 and included patients receiving TSH suppression therapy. A self-designed questionnaire was employed to collect their demographic characteristics and KAP. The analysis included 528 valid questionnaires.

**Results:**

The mean knowledge, attitude, and practice scores were 11.66 ± 5.82 (possible range: 0-22), 33.31 ± 4.97 (possible range: 0-40), and 24.46 ± 3.46 (possible range: 0-34), respectively, indicating poor knowledge, favorable attitudes, and moderate practice. The knowledge scores were correlated to the attitude (r=0.399, P<0.001) and practice (r=0.401, P<0.001) scores, while the attitude scores were correlated to the practice scores (r=0.512, P<0.001). The structural equation modeling revealed that knowledge directly influenced attitude (β=0.34, P<0.001) and practice (β=0.13, P<0.001) and indirectly influenced practice through attitude (β=0.10, P<0.001).

**Conclusions:**

Patients receiving TSH suppression therapy in Zhanjiang demonstrated poor knowledge, favorable attitudes, and moderate practices toward TSH suppression therapy.

## Introduction

Thyroid cancer is a cancer growing from the parenchymal cells in the thyroid (1). It is the 11^th^ most common cancer worldwide, with approximately 586,202 new cases and 43,646 associated deaths reported in 2020 (2). Among thyroid cancers, papillary thyroid cancer (PTC) is the predominant type, constituting around 80% of all cases (3). PTC exhibits a broad spectrum of biological behaviors, ranging from indolent, nonprogressive nodules within the thyroid gland to aggressive metastatic cancers. Fortunately, PTC generally boasts an excellent prognosis, particularly with meticulous management (3, 4). Primary treatment for thyroid cancer typically involves surgery, serving as the frontline approach for the majority of patients. Additionally, radioactive iodine (RAI) therapy has been conventionally employed as adjuvant treatment to enhance disease-free survival (4–8). Following thyroid surgery and RAI remnant ablation, thyroid-stimulating hormone (TSH) suppression therapy can be performed in selected high-risk patients (e.g., incomplete resection, metastatic disease, and lymph nodes >3 cm). Low-risk patients may be also considered for TSH suppression; however, the treatment targets differ from those for high-risk patients (4, 6, 7). Thyroid cancer cells rely on TSH for growth (9). Hence, TSH suppression utilizes supraphysiologic doses of levothyroxine to suppress TSH production, potentially diminishing the risk of recurrence and preventing adverse events (6). TSH suppression therapy plays a crucial role in optimizing post-treatment outcomes and maintaining the favorable prognosis associated with well-managed thyroid cancer (6).

However, TSH suppression therapy, a daily oral medication taken at home, is not without side effects, encompassing osteoporosis, fractures, cardiovascular risks, and mood disorders (9–12). Given its importance, patient cooperation becomes paramount, and non-compliance could significantly impact treatment outcomes and prognosis (13, 14). A comprehensive understanding of the risks associated with not adhering to TSH suppression therapy, potential side effects, and strategies to mitigate these risks is crucial for fostering positive attitudes and practices toward the therapy. To address this need, Knowledge, Attitude, and Practice (KAP) surveys are instrumental. These surveys help pinpoint knowledge gaps, misconceptions, and misunderstandings that may act as barriers to the optimal execution of a prescribed action within a specific population (15, 16). Conducting KAP studies is imperative for identifying factors that could potentially influence patient prognosis. Despite the critical nature of TSH suppression therapy, no prior studies have explored the KAP of individuals towards this therapy. Nevertheless, some studies have highlighted inadequate KAP concerning thyroid cancer in general (17–20).

Therefore, this study aimed to investigate the KAP toward TSH suppression therapy among postoperative patients with thyroid cancer treated with TSH suppression therapy.

## Methods

### Study design and participants

This cross-sectional study was conducted at our Hospital between March 2023 and October 2023. The inclusion criteria were 1) patients who underwent thyroid cancer surgery and 2) received postoperative TSH suppression therapy. The exclusion criteria were 1) impaired consciousness, 2) a history of mental illness, 3) incomplete or missing clinical data or pathology reports, or 4) <18 years of age. The study was approved by the ethics committee of our Hospital, and written informed consent was obtained from all participants before they completed the survey.

### Questionnaire

The questionnaire was designed by the investigators based on the *“Consensus of Chinese Experts on Postoperative Management of Differentiated Thyroid Cancer* (2020)*,” “Guidelines for Diagnosis and Treatment of Differentiated Thyroid Cancer by the Chinese Society of Clinical Oncology (2021),”* and the *“Clinical Practice Guidelines for the Diagnosis and Treatment of Thyroid Nodules by the American Association of Clinical Endocrinologists”* (5). The questionnaire was revised for content validity by two experts with >20 years of experience in thyroid cancer. Unclear questions were revised. Then, a pilot study with a small sample size (36 participants) was conducted, revealing a reliability coefficient (Cronbach’s α) of 0.915. In order to address face value, the participants were also asked to indicate any question or statement that was hard to understand. None of the questions were considered difficult to understand after explanation, suggesting acceptable face validity. Given the limited sample size of the pilot study, construct validity was assessed using confirmatory factor analysis based on data from the formal survey (**Supplementary Figure S1**), with all fit indices indicating good construct validity (**Supplementary Table S1**).

The final questionnaire was in Chinese and encompassed four dimensions comprising 50 items: 17 for demographic characteristics, 16 for knowledge, 10 for attitude, and 7 for practice. In the knowledge dimension, items with correct answers received 1 point, while incorrect or unclear responses received 0 points. The scoring for the comprehension of knowledge was 2 points for correct understanding, 1 point for partial comprehension, and 0 points for no comprehension. The total scores in the knowledge dimension ranged from 0 to 22 points. The attitude dimension primarily utilized a 5-point Likert scale, ranging from very positive (4 points) to very negative (0 points), with a score range of 0 to 40 points. Items A2, A5-A8, and A10 were positively scored, while items A1, A3, A4, and A9 were negatively scored. For the practice dimension, item P1 was scored 4 points for “yes” and 0 points for “no”, while items P2 to P7 used a 5-point Likert scale for positive scoring. The total practice score was 0 to 34. Other unscorable items were treated as categorical variables.

Participants’ overall knowledge, attitude, and practice scores were categorized using a modified Bloom’s criteria cutoff point. Participants who scored 80%-100% of the total score had good knowledge, positive attitude, and appropriate practice, respectively, while 60%-79% was considered moderate, and <60% indicated poor knowledge, negative attitude, and inappropriate practice, respectively (21).

Questionnaires were created using the Questionnaire Star platform on WeChat. A QR code was generated and advertised to the patients through leaflets and posters in the waiting room and by the nurses and physicians. Questionnaires answered using all the same options (e.g., all last options) or logic issues (e.g., impossible age) were considered invalid.

### Sample size

Regarding the sample size, the minimal sample size was estimated using Cochran’s sample size formula for survey studies (22):


$$n=\frac{Z^{2}\times p\left(1-p\right)}{e^{2}}$$

where *Z^2^* is the confidence coefficient, p is the proportion, and e is the margin of error. The sample size is maximized when *p*=0.5. A 95% confidence interval involves a *Z*-value of 1.96. Precision was assumed at 5%. Hence, a minimum of 384 participants were needed.

### Statistical analysis

Statistical analysis was conducted using R 4.3.1. Continuous variables following a normal distribution (according to the Kolmogorov-Smirnov test) were expressed as means ± standard deviations and compared using Student’s t-test or one-way analysis of variance (ANOVA). The categorical data were presented as n (%) and analyzed using the chi-squared test. The Pearson correlation analysis was used to assess the correlations between the three KAP dimensions. A structural equation modeling (SEM) analysis was performed to validate the following hypotheses: 1) knowledge influences attitudes; 2) attitudes influence practices; 3) knowledge influences practices.

## Results

### Characteristics of the participants

In total, 578 questionnaires were collected; among these, 1 had too short response time, 28 exhibited logical conflicts, and 21 displayed distinct response patterns. Therefore, 528 valid questionnaires were included for analysis. The participants were 44.48 ± 11.91 years old. Most participants were female (80.11%), married (88.07%), urban residents (52.08%), with college or above education (40.34%), employed (57.39%), with a monthly income of 2,000-4,999 Yuan (37.31%), with health insurance (94.89%), non-smoking (90.91%), non-drinking (90.34%), <2 years since thyroid cancer diagnosis (72.92%), <2 years since thyroid cancer surgery (75.00%), without lymph node metastasis (54.55%), non-metastatic (83.33%), and without comorbidities (75.76%) (**Table 1**).

**Table 1:** Characteristics of the participants and KAP scores.

	n (%)	Knowledge		Attitude		Practice	
		Score	P	Score	P	Score	P
All	528 (100)	11.66 ± 5.82		33.31 ± 4.97		24.46 ± 3.46	
Age	44.48 ± 11.91						
Gender			0.242		0.671		0.073
Male	105 (19.89)	12.37 ± 5.89		33.72 ± 4.72		25.10 ± 3.21	
Female	423 (80.11)	11.49 ± 5.80		33.21 ± 5.03		24.30 ± 3.50	
Marital Status			0.050		0.136		0.742
Unmarried	63 (11.93)	13.06 ± 5.54		32.70 ± 4.23		24.59 ± 3.37	
Married	465 (88.07)	11.47 ± 5.84		33.39 ± 5.06		24.44 ± 3.47	
Residence			0.002		0.891		0.002
Rural	221 (41.86)	11.00 ± 5.88		33.22 ± 5.25		24.91 ± 3.20	
Urban	275 (52.08)	12.45 ± 5.65		33.42 ± 4.77		24.35 ± 3.43	
Suburban	32 (6.06)	9.41 ± 5.85		33.00 ± 4.79		22.25 ± 4.50	
Education			<0.001		0.030		0.633
Primary school and below	73 (13.83)	10.55 ± 6.03		31.52 ± 5.98		24.08 ± 3.62	
Junior high school	154 (29.17)	9.99 ± 5.79		33.19 ± 5.00		24.60 ± 3.54	
High school/technical secondary school	88 (16.67)	11.27 ± 5.40		33.64 ± 4.75		24.23 ± 3.58	
College and above	213 (40.34)	13.42 ± 5.48		33.87 ± 4.53		24.59 ± 3.30	
Employment			0.002		0.02		0.89
Employed	303 (57.39)	12.33 ± 5.63		33.66 ± 4.66		24.57 ± 3.26	
Unemployed	133 (25.19)	10.20 ± 5.80		32.12 ± 5.57		24.19 ± 3.89	
Other	92 (17.42)	11.60 ± 6.12		33.87 ± 4.81		24.50 ± 3.47	
Monthly income, Yuan			<0.001		0.082		0.798
<2,000	149 (28.22)	9.356 ± 6.14		32.42 ± 5.49		24.21 ± 3.71	
2,000-4,999	197 (37.31)	11.89 ± 5.27		33.47 ± 4.78		24.55 ± 3.16	
5,000-9,999	140 (26.52)	13.06 ± 5.60		33.61 ± 4.75		24.48 ± 3.67	
≥10,000	42 (7.95)	14.12 ± 5.26		34.69 ± 4.22		24.88 ± 3.23	
Health insurance type							
Only social medical insurance			0.374		0.815		0.530
No	99 (18.75)	12.10 ± 6.03		33.17 ± 5.58		24.58 ± 3.63	
Yes	429 (81.25)	11.56 ± 5.77		33.34 ± 4.83		24.43 ± 3.42	
Only commercial medical insurance			0.372		0.722		0.550
No	522 (98.86)	11.69 ± 5.80		33.32 ± 4.95		24.46 ± 3.47	
Yes	6 (1.14)	9.50 ± 7.69		32.00 ± 6.69		24.17 ± 1.94	
Both social and commercial medical insurance			0.006		0.455		0.930
No	448 (84.85)	11.37 ± 5.80		33.26 ± 4.94		24.48 ± 3.42	
Yes	80 (15.15)	13.33 ± 5.67		33.58 ± 5.18		24.35 ± 3.71	
No insurance			0.041		0.307		0.398
No	501 (94.89)	11.79 ± 5.79		33.38 ± 4.92		24.44 ± 3.43	
Yes	27 (5.11)	9.22 ± 6.03		32.07 ± 5.86		24.78 ± 3.95	
Smoking status			0.982		0.390		0.710
No	480 (90.91)	11.67 ± 5.82		33.27 ± 4.93		24.45 ± 3.44	
Yes	48 (9.09)	11.63 ± 5.85		33.69 ± 5.38		24.54 ± 3.64	
Alcohol consumption			0.233		0.632		0.372
No	477 (90.34)	11.57 ± 5.75		33.26 ± 5.03		24.44 ± 3.41	
Yes	51 (9.66)	12.49 ± 6.46		33.76 ± 4.43		24.65 ± 3.93	
Time since thyroid cancer diagnosis			0.565		0.348		<0.001
<3 months	137 (25.95)	11.95 ± 6.08		34.01 ± 4.91		25.30 ± 3.23	
3-6 months (<6 months)	68 (12.88)	12.15 ± 6.01		33.10 ± 5.46		25.06 ± 2.98	
6-12 months (<12 months)	82 (15.53)	10.98 ± 5.78		33.01 ± 5.12		23.90 ± 3.41	
1-2 years (<2 years)	98 (18.56)	12.08 ± 5.12		33.12 ± 4.56		24.43 ± 3.41	
≥2 years	143 (27.08)	11.27 ± 5.96		33.03 ± 4.96		23.71 ± 3.75	
Time since thyroid cancer surgery			0.636		0.030		<0.001
<3 months	171 (32.39)	11.85 ± 6.18		34.01 ± 5.02		25.35 ± 3.02	
3-6 months (<6 months)	68 (12.88)	11.76 ± 5.53		33.87 ± 4.93		24.76 ± 3.22	
6-12 months (<12 months)	79 (14.96)	10.82 ± 5.79		32.29 ± 5.12		23.82 ± 3.67	
1-2 years (<2 years)	78 (14.77)	12.27 ± 5.15		32.63 ± 4.80		24.12 ± 3.39	
≥2 years	132 (25.00)	11.51 ± 5.90		33.12 ± 4.83		23.73 ± 3.77	
Other thyroid cancer treatment methods (apart from surgery)							
None			0.220		0.304		<0.001
No	344 (65.15)	11.45 ± 5.60		33.14 ± 5.03		24.09 ± 3.45	
Yes	184 (34.85)	12.05 ± 6.21		33.63 ± 4.86		25.15 ± 3.37	
Physical ablation			0.024		0.284		0.349
No	519 (98.30)	11.59 ± 5.810		33.28 ± 4.965		24.44 ± 3.469	
Yes	9 (1.70)	16.00 ± 4.899		34.89 ± 5.302		25.67 ± 2.646	
Thyroxine tablets (Euthyrox)			0.151		0.320		<0.001
No	202 (38.26)	12.08 ± 6.25		33.61 ± 4.81		25.06 ± 3.31	
Yes	326 (61.74)	11.40 ± 5.53		33.12 ± 5.07		24.09 ± 3.50	
Iodine-131 treatment			0.132		0.719		0.691
No	479 (90.72)	11.53 ± 5.81		33.27 ± 5.01		24.47 ± 3.47	
Yes	49 (9.28)	12.92 ± 5.80		33.65 ± 4.64		24.33 ± 3.36	
Radiotherapy			0.014		0.416		0.238
No	525 (99.43)	11.71 ± 5.80		33.32 ± 4.98		24.47 ± 3.46	
Yes	3 (0.57)	3.00 ± 2.00		32.00 ± 1.00		22.67 ± 3.06	
Traditional Chinese medicine			0.194		0.751		0.732
No	525 (99.43)	11.64 ± 5.828		33.30 ± 4.982		24.46 ± 3.464	
Yes	3 (0.57)	15.67 ± 1.528		34.67 ± 2.082		25.33 ± 2.517	
Other treatment							
No	527 (99.81)						
Yes	1 (0.19)						
Lymph node metastasis in thyroid cancer			<0.001		0.039		0.044
Yes	151 (28.60)	13.18 ± 5.81		33.70 ± 4.55		25.01 ± 3.11	
No	288 (54.55)	11.66 ± 5.57		33.55 ± 4.88		24.44 ± 3.40	
Unknown	89 (16.86)	9.11 ± 5.80		31.87 ± 5.69		23.60 ± 4.03	
Metastasis of thyroid cancer to other organs			<0.001		0.005		0.020
Yes	9 (1.70)	12.67 ± 7.09		30.56 ± 6.77		23.67 ± 2.69	
No	440 (83.33)	12.23 ± 5.63		33.66 ± 4.75		24.68 ± 3.31	
Unknown	79 (14.96)	8.37 ± 5.67		31.65 ± 5.54		23.35 ± 4.09	
Direct relatives who underwent thyroid cancer surgery			0.012		0.231		0.403
Yes	70 (13.26)	12.64 ± 5.70		32.57 ± 5.17		23.94 ± 3.69	
No	435 (82.39)	11.69 ± 5.80		33.48 ± 4.91		24.55 ± 3.43	
Unknown	23 (4.36)	8.22 ± 5.76		32.39 ± 5.43		24.39 ± 3.17	
Other underlying diseases			0.105		0.927		0.983
No	400 (75.76)	11.89 ± 5.95		33.35 ± 4.90		24.42 ± 3.56	
Yes	128 (24.24)	10.96 ± 5.34		33.20 ± 5.20		24.58 ± 3.14	

### Knowledge, attitude, and practice

The mean knowledge score was 11.66 ± 5.82 (possible range: 0-22), indicating poor knowledge. Higher knowledge scores were observed in unmarried participants (P=0.050), urban residents (P=0.002), with higher education (P<0.001), employed (P=0.002), with higher income (P<0.001), without insurance (P=0.041), who underwent physical ablation (P=0.024), without radiotherapy (P=0.014), with lymph node metastasis (P<0.001), with distant metastasis (P<0.001), and with direct relatives with thyroid cancer surgery (P=0.012) (**Table 1**). The knowledge items with poor scores included K1 (41.67%; *“The 5-year survival rate of thyroid cancer can reach over 80%, but there is still a risk of death”*), K2 (8.52%; *“The prognosis of thyroid cancer is mainly related to pathological type, age, tumor size, etc., with undifferentiated cancer having a better prognosis and papillary cancer having the worst prognosis”*), K7-1 (46.02%; *“Use medication to prevent nausea, vomiting, and other high-risk actions”*), K7-4 (17.05%; *“Practice voice exercises and singing”*), K8 (20.08%; *“Thyroid cancer is a thyrotropin (TSH)-dependent tumor, and postoperative TSH levels are related to tumor recurrence, metastasis, and death”*), K9 (19.51%; *“After thyroid cancer surgery, timely TSH suppression therapy should be initiated, and the initial suppression target is based on the initial risk stratification for recurrence”*), K10 (51.33%; *“L-T4 oral preparation (levothyroxine tablets, i.e., Euthyrox) is the preferred choice for TSH suppression therapy”*), K11 (26.52%; *“There is a certain risk of recurrence after thyroid cancer surgery. For patients stratified as high risk for recurrence, iodine-131 treatment is recommended”*), K12 (44.13%; *“Patients after thyroid cancer surgery should pay attention to a balanced and sensible diet, limit alcohol intake, moderately restrict sugar consumption, and maintain a healthy weight for life”*), and K13 (32.95%; *“Appropriate exercise rehabilitation measures can significantly improve patient anxiety and depression, enhance their quality of life. It is recommended to engage in aerobic training three times a week at moderate intensity, lasting at least 12 weeks, or aerobic combined with resistance training twice a week, lasting at least 6 weeks”*) (**Supplementary Table S2**).

The mean attitude score was 33.31 ± 4.97 (possible range: 0-40), indicating favorable attitudes. Higher attitude scores were observed with higher education (P=0.030), employed (P=0.020), with shorter time since thyroid cancer surgery (P=0.030), with lymph node metastasis (P=0.039), and with distant metastasis (P=0.005) (**Table 1**). All the attitude items demonstrated a positive attitude (**Supplementary Table S3**).

The mean practice score was 24.46 ± 3.46 (possible range: 0-34, 71.94%), indicating moderate practice. Higher practice scores were observed with rural and urban residents (P=0.002), with shorter time from thyroid cancer diagnosis (P<0.001), with shorter time since thyroid cancer surgery (P<0.001), with other treatment methods (P<0.001), without thyroxine tablets (P<0.001), with lymph node metastasis (P=0.044), and with distant metastasis (P=0.020) (**Table 1**). Most items in the practice dimension demonstrated proactive practice, but some practice showed less favorable results, including *“P4: You maintain a balanced and healthy diet as the doctor recommends after thyroid cancer surgery,” “P5: You engaged in appropriate neck function exercises early on after thyroid cancer surgery,”* and *“P6: You started early mobilization and, with the doctor’s approval, engaged in suitable full-body rehabilitation exercises after thyroid cancer surgery”* (**Supplementary Table S4**).

### Correlations among knowledge, attitude, and practice

The knowledge scores were correlated to the attitude (r=0.399, P<0.001) and practice (r=0.401, P<0.001) scores, while the attitude scores were correlated to the practice scores (r=0.512, P<0.001) (**Table 2**). The SEM demonstrated knowledge directly influenced attitude (β=0.34, P<0.001) and practice (β=0.13, P<0.001) and also indirectly influenced practice through attitude (β=0.10, P<0.001) (**Table 3**).

**Table 2:** Correlation analysis.

	Knowledge	Attitude	Practice
Knowledge	1.000		
Attitude	0.399 (P<0.001)	1.000	
Practice	0.401 (P<0.001)	0.512 (P<0.001)	1.000

**Table 3:** Structural equation modeling.

Model paths	Total effects		Direct effect		Indirect effect	
	β (95% CI)	P	β (95% CI)	P	β (95% CI)	P
Attitude <- Knowledge	0.34 (0.27,0.41)	<0.001	0.34 (0.27,0.41)	<0.001		
Practice <- Attitude	0.29 (0.24,0.35)	<0.001	0.29 (0.24,0.35)	<0.001		
Practice <- Knowledge	0.23 (0.19,0.28)	<0.001	0.13 (0.08,0.18)	<0.001	0.10 (0.07,0.12)	<0.001

## Discussion

This study suggests that patients with thyroid cancer undergoing TSH suppression therapy in Zhanjiang exhibited poor knowledge, favorable attitudes, and moderate practices concerning TSH suppression therapy. The findings have the potential to pinpoint specific knowledge gaps that, when addressed through targeted education, may lead to improvements in both attitudes and practices related to TSH suppression therapy.

The treatment of thyroid cancer is multidisciplinary (4–8). Many procedures that aim to improve prognosis (e.g., surgery and RAI therapy) are performed or given at the hospital as long as the patients are present at their appointments. On the other hand, TSH suppression therapy is given orally at home, implying that patient adherence to treatment is central to optimizing prognosis (13, 14). Treatment adherence depends upon the patient’s understanding of the treatment and its side effects and upon the patient’s attitude toward the treatment, which will together impact the practice of taking the treatment or not. Therefore, a proper patient KAP toward the treatments taken at home is primordial (13, 14).

In the present study, most participants were female and in mid-adulthood, consistent with the epidemiology of thyroid cancer (8). A higher socioeconomic status (education and income) was associated with higher KAP scores. Indeed, the socioeconomic status is associated with better health literacy (23). Participants with specific treatments also had a higher KAP, probably because more information was given in the setting of these special treatments. Participants with a shorter time from diagnosis or surgery also had better KAP, possibly because the information was fresher in their memory.

The present study showed that the patients on TSH suppression therapy had poor knowledge but favorable attitudes and moderate practice toward their TSH suppression therapy. It suggests that they generally follow the physicians’ advice without understanding them. Still, poor knowledge could pose some issues regarding safety, especially regarding which side effects to look for, which examinations should be performed to monitor the development of side effects, and how to prevent them. No previous studies examined the KAP toward TSH suppression therapy, but some previous studies examined the KAP toward thyroid cancer in general and also demonstrated poor knowledge (17–20). Li et al. (17) reported that patients in South China had poor knowledge but moderate attitudes and proactive practice toward thyroid nodules and thyroid cancer. Iqbal et al. (18) showed that Pakistani university students had a poor knowledge of the factors associated with thyroid cancers and of the early signs and symptoms to look for. Similar results were reported in Saudi Arabia (19, 20, 24). A meta-analysis of 10 studies (two from Saudi Arabia and one each from Malaysia, China, Korea, Israel, Pakistan, The Netherlands, United States of America, and Yemen) showed good awareness but poor knowledge and perception of thyroid cancer and risk factors (25). Patients with hypothyroidism in Delhi have poor KAP toward their treatment and low adherence (26).

In the present study, the KAP dimensions were positively correlated to each other. In addition, in the SEM analysis, knowledge directly influenced attitude, attitude directly influenced practice, and knowledge directly and indirectly influenced practice. Therefore, improving knowledge should also improve attitude and practice. Indeed, according to the KAP theory, knowledge is the basis for practice, while attitude is the energy-driving practice (15, 16). Educational interventions should be designed and implemented to enhance the knowledge of patients with thyroid cancer regarding TSH suppression therapy. Specifically, there is a need to improve understanding of the natural history and prognosis of thyroid cancer, the risk of postoperative bleeding, the role of TSH in thyroid cancer, TSH suppression therapy, thyroid cancer recurrence, and lifestyle habits. Educational interventions addressing these items could help improve patient self-management, which could translate into better prognosis, quality of life, and quality of survivorship. By addressing these areas, patient practices can be positively influenced, particularly in relation to diet, functional exercises, and rehabilitation exercises. Such education could be provided face-to-face by healthcare providers, as well as through pamphlets, videos, and interactive web platforms. Still, some KAP aspects were not evaluated in the present study, e.g., how to take levothyroxine, trusting medical treatment, knowledge about the different forms of levothyroxine, and assessing the knowledge of the risk of self-adjusting levothyroxine. Indeed, the timing of taking the levothyroxine, the knowledge of the different forms of levothyroxine, and the risks associated with self-adjustment are important points since they can influence the TSH levels (27–29). These aspects should be examined in future studies.

This study had limitations. It was a single-center study, resulting in a relatively small sample size and limiting generalizability since all patients were from the same geographical area. The study was cross-sectional, and the data represent a single point in time. Still, the data could be used as a historical baseline for future intervention studies. In addition, the questionnaire was designed by local investigators based on local practices and policies, limiting its exportability to other centers and the generalizability of the data. Furthermore, the data were cross-sectional and self-reported (15, 16, 30). Hence, precise clinical data (e.g., TSH levels or thyroxine dosage) were not collected due to worries about the validity of such self-reported data. Indeed, only 61% of the participants on TSH suppressing therapy reported receiving thyroxine tablets, possibly because the patient did not know the name of the drug, consistent with the low knowledge score. In addition, 90% of the participants reported not having received radioiodine treatment. These examples highlight the potential bias of self-reported medical data and also support the finding of participants’ low knowledge. Therefore, some aspects of treatment adherence were evaluated in the practice dimension, but adherence was not formally evaluated using clinical outcomes or a formally validated questionnaire on adherence. Moreover, the questionnaire did not cover all KAP aspects. Future studies should consider factors such as the timing of levothyroxine intake, knowledge of different forms of levothyroxine, and the risks associated with self-adjustment. Finally, all KAP studies are at risk of the social desirability bias, which entails that patients could be tempted to answer what they know they should be doing instead of what they are actually doing (31, 32). Still, since knowledge was poor and attitude and practice had better scores, the risk of the social desirability bias should be low.

In conclusion, patients with thyroid cancer undergoing TSH suppression therapy in Zhanjiang demonstrated poor knowledge, favorable attitudes, and moderate practices regarding TSH suppression therapy. The study successfully identified specific knowledge gaps that could be addressed through education to enhance attitudes and practices related to TSH suppression therapy. It is important to note that adherence and prognosis were not specifically examined in the present study. However, the potential impact of education on adherence and prognosis should be evaluated in future studies.

## Data Availability

The original contributions presented in the study are included in the article/**Supplementary Material**. Further inquiries can be directed to the corresponding author.

## Supplementary material

Supplementary Figure 1 Confirmatory factor analysis of the questionnaire.
