# Supplementary figures and images for: Knowledge, attitudes, and practices regarding the postoperative management and TSH suppression therapy among patients with thyroid cancer
Source: Front Oncol. 2025 Mar 11;15:1441726. doi: 10.3389/fonc.2025.1441726 (PMC11933125; doi:10.3389/fonc.2025.1441726)

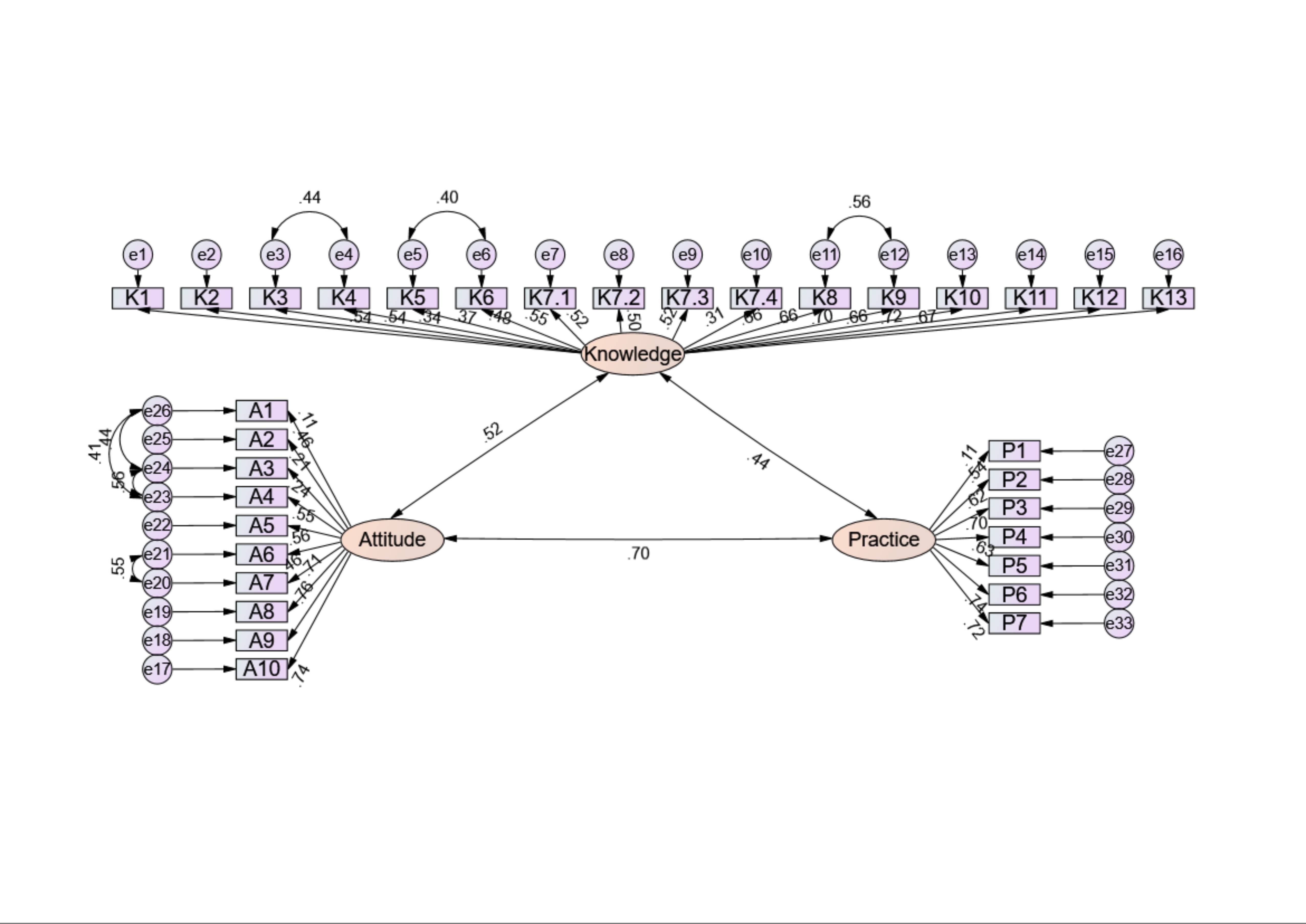

Supplement: Supplementary Figure 1 — Confirmatory factor analysis of the questionnaire. [file Image1.jpeg]
