# Supplementary material for: Knowledge, attitudes, and practices regarding the postoperative management and TSH suppression therapy among patients with thyroid cancer
Source: Front Oncol. 2025 Mar 11;15:1441726. doi: 10.3389/fonc.2025.1441726 (PMC11933125; doi:10.3389/fonc.2025.1441726)
Supplement: Supplementary file 2 [file Table1.docx]

**Supplementary Table S1.** Confirmatory factor analysis of the questionnaire.

| Indicators | Reference | Actual |
| --- | --- | --- |
| CMIN/DF | 1-3: excellent; 3-5: good | 3.428 |
| RMSEA | <0.08: good | 0.068 |
| IFI | >0.8: good | 0.822 |
| TLI | >0.8: good | 0.805 |
| CFI | >0.8: good | 0.821 |

CMIN/DF: Minimum Discrepancy Function by Degrees of Freedom divided; RMSEA: Root Mean Square Error of Approximation; IFI: incremental fit index; TLI: Tucker-Lewis index; CFI: comparative fit model.
