# Supplementary material for: Knowledge, attitudes, and practices regarding the postoperative management and TSH suppression therapy among patients with thyroid cancer
Source: Front Oncol. 2025 Mar 11;15:1441726. doi: 10.3389/fonc.2025.1441726 (PMC11933125; doi:10.3389/fonc.2025.1441726)
Supplement: Supplementary file 3 [file Table2.docx]

**Supplementary Table S2.** Participant knowledge distribution

|  | Correct | Incorrect | Unknown |
| --- | --- | --- | --- |
| 1. The 5-year survival rate of thyroid cancer can reach over 80%, but there is still a risk of death. | 41.67 | 4.17 | 54.17 |
| 2. The prognosis of thyroid cancer is mainly related to pathological type, age, tumor size, etc., with undifferentiated cancer having a better prognosis and papillary cancer having the worst prognosis. | 8.52 | 33.33 | 58.14 |
| 3. For most thyroid cancer patients, it is advisable to be conscious and comfortable or engage in moderate bed activities after surgery, and early mobilization is recommended. | 65.91 | 14.39 | 19.70 |
| 4. For most thyroid cancer patients, after fully waking up from surgery, they can try drinking water. If there is no discomfort, they can gradually transition to a semi-liquid or regular diet. | 72.35 | 8.71 | 18.94 |
| 5. Within the first month after thyroid cancer surgery, avoid vigorously rubbing the incision, refrain from strenuous activities, and ensure the incision stays dry after bathing. | 84.28 | 1.52 | 14.20 |
| 6. Early postoperative neck exercises, such as moderate head turning, shoulder circling, and raising and lowering the arms, can alleviate discomfort for most thyroid cancer patients, but strenuous activities should be avoided. | 75.95 | 2.46 | 21.59 |
| 7. Postoperative bleeding in thyroid cancer is a rare but life-threatening complication, with most cases occurring within 24 hours after surgery. Which of the following measures are beneficial for preventing postoperative bleeding: | | | |
| 7-1. Use medication to prevent nausea, vomiting, and other high-risk actions. | 46.02 | 6.06 | 47.92 |
| 7-2. Avoid vigorous neck movements. | 74.43 | 3.60 | 21.97 |
| 7-3. Control blood pressure. | 69.32 | 1.89 | 28.79 |
| 7-4. Practice voice exercises and singing. | 17.05 | 52.84 | 30.11 |
|  | Understand | Partially understand | Do not understand |
| 8. Thyroid cancer is a thyrotropin (TSH)-dependent tumor and postoperative TSH levels are related to tumor recurrence, metastasis, and death. | 20.08 | 24.62 | 55.30 |
| 9. After thyroid cancer surgery, timely TSH suppression therapy should be initiated, and the initial suppression target is based on the initial risk stratification for recurrence. | 19.51 | 24.05 | 56.44 |
| 10. L-T4 oral preparation (levothyroxine tablets, i.e., Euthyrox) is preferred for TSH suppression therapy. | 51.33 | 26.33 | 22.35 |
| 11. There is a certain risk of recurrence after thyroid cancer surgery. For patients stratified as high risk for recurrence, iodine-131 treatment is recommended. | 26.52 | 24.24 | 49.24 |
| 12. Patients after thyroid cancer surgery should pay attention to a balanced and sensible diet, limit alcohol intake, moderately restrict sugar consumption, and maintain a healthy weight for life. | 44.13 | 31.25 | 24.62 |
| 13. Appropriate exercise rehabilitation measures can significantly improve patient anxiety and depression and enhance their quality of life. Engaging in aerobic training three times a week at moderate intensity, lasting at least 12 weeks, or aerobic combined with resistance training twice a week, lasting at least 6 weeks, is recommended. | 32.95 | 30.68 | 36.36 |
