# Supplementary material for: Knowledge, attitudes, and practices regarding the postoperative management and TSH suppression therapy among patients with thyroid cancer
Source: Front Oncol. 2025 Mar 11;15:1441726. doi: 10.3389/fonc.2025.1441726 (PMC11933125; doi:10.3389/fonc.2025.1441726)
Supplement: Supplementary file 4 [file Table3.docx]

**Supplementary Table S3.** Participant attitude distribution

|  | Strongly agree | Agree | Neutral | Disagree | Strongly disagree |
| --- | --- | --- | --- | --- | --- |
| 1. You believe that the survival rate for thyroid cancer is very high, and once surgery is done, there is no need to worry about it anymore. | 5.11 | 4.55 | 12.88 | 24.05 | 53.41 |
| 2. You believe that receiving standardized TSH suppression therapy after thyroid cancer surgery is very important. | 49.43 | 25.57 | 21.02 | 1.89 | 2.08 |
| 3. You believe that undergoing surgery for thyroid cancer is already a cure, and there is no need to continue taking medication. | 5.11 | 5.11 | 10.61 | 19.89 | 59.28 |
| 4. You believe that the prognosis for thyroid cancer is good and there is no need for long-term follow-up after surgery. | 5.49 | 7.20 | 8.33 | 22.92 | 56.06 |
|  | Very confident | Confident | Neutral | Unconfident | Very unconfident |
| 5. Confidence in treating and overcoming thyroid cancer: | 55.49 | 32.20 | 11.17 | 0.57 | 0.57 |
|  | Highly value | Value | Neutral | Consider less important | Do not value at all |
| 6. After thyroid cancer surgery, you place a high importance on a balanced and reasonable diet. | 48.11 | 36.36 | 14.02 | 0.95 | 0.57 |
| 7. After thyroid cancer surgery, you place a high importance on appropriate exercise. | 37.88 | 34.09 | 26.14 | 0.76 | 1.14 |
|  | Strongly agree | Agree | Neutral | Disagree | Strongly disagree |
| 8. You believe that your communication with the doctor is effective and sufficient. | 66.67 | 26.70 | 5.49 | 0.57 | 0.57 |
| 9. You believe educating the public about the postoperative management of thyroid cancer patients is necessary. | 67.99 | 25.38 | 5.68 | 0.38 | 0.57 |
| 10. You believe promoting proactive screening for thyroid nodules and thyroid cancer in the general public is important. | 67.80 | 24.62 | 7.01 | 0.38 | 0.19 |
