# Supplementary material for: Knowledge, attitudes, and practices regarding the postoperative management and TSH suppression therapy among patients with thyroid cancer
Source: Front Oncol. 2025 Mar 11;15:1441726. doi: 10.3389/fonc.2025.1441726 (PMC11933125; doi:10.3389/fonc.2025.1441726)
Supplement: Supplementary file 5 [file Table4.docx]

**Supplementary Table S4.** Distribution of participant practice

|  | Yes | No |  |  |  |
| --- | --- | --- | --- | --- | --- |
| 1. After thyroid cancer surgery, did you follow the doctor’s advice to undergo TSH suppression therapy? | 91.86 | 8.14 |  |  |  |
|  | Highly consistent | Consistent | Neutral | Not very consistent | Not consistent at all |
| 2. You take the prescribed medication daily, as directed, after thyroid cancer surgery. | 84.66 | 12.69 | 2.27 | 0.38 | 0.00 |
| 3. You attend regular follow-up appointments as advised by the doctor after thyroid cancer surgery. | 76.52 | 18.75 | 4.55 | 0.19 | 0.00 |
| 4. You maintain a balanced and healthy diet as the doctor recommends after thyroid cancer surgery. | 52.84 | 30.68 | 15.34 | 0.76 | 0.38 |
| 5. You engaged in appropriate neck function exercises early on after thyroid cancer surgery. | 42.23 | 28.22 | 23.67 | 3.22 | 2.65 |
| 6. You started early mobilization and, with the doctor’s approval, engaged in suitable full-body rehabilitation exercises after thyroid cancer surgery. | 49.81 | 30.11 | 18.75 | 0.57 | 0.76 |
| 7. You proactively seek advice from the doctor regarding postoperative considerations for thyroid cancer. | 67.23 | 24.81 | 7.58 | 0.19 | 0.19 |
